# G protein–coupled estrogen receptor: a promising therapeutic target for aldosterone-induced hypertension

**DOI:** 10.3389/fendo.2023.1226458

**Published:** 2023-08-17

**Authors:** Xuehan Li, Wenlong Kuang, Zhihua Qiu, Zihua Zhou

**Affiliations:** ^1^ Department of Cardiology, Union Hospital, Tongji Medical College, Huazhong University of Science and Technology, Wuhan, China; ^2^ Hubei Key Laboratory of Biological Targeted Therapy, Union Hospital, Tongji Medical College, Huazhong University of Science and Technology, Wuhan, China; ^3^ Hubei Engineering Research Center for Immunological Diagnosis and Therapy of Cardiovascular Diseases, Union Hospital, Tongji Medical College, Huazhong University of Science and Technology, Wuhan, China

**Keywords:** GPER, aldosterone, nongenomic pathways, hypertension, new target

## Abstract

Aldosterone is one of the most essential hormones synthesized by the adrenal gland because it regulates water and electrolyte balance. G protein–coupled estrogen receptor (GPER) is a newly discovered aldosterone receptor, which is proposed to mediate the non-genomic pathways of aldosterone while the hormone simultaneously interacts with mineralocorticoid receptor. In contrast to its cardio-protective role in postmenopausal women via its interaction with estrogen, GPER seems to trigger vasoconstriction effects and can further induce water and sodium retention in the presence of aldosterone, indicating two entirely different binding sites and effects for estrogen and aldosterone. Accumulating evidence also points to a role of aldosterone in mediating hypertension and its risk factors via the interaction with GPER. Therefore, with this review, we aimed to summarize the research on these interactions to help (1) elucidate the role of GPER activated by aldosterone in the blood vessels, heart, and kidney; (2) compare the non-genomic actions between aldosterone and estrogen mediated by GPER; and (3) address the potential of GPER as a new promising therapeutic target for aldosterone-induced hypertension.

## 1 Introduction

Hypertension is considered one of the most common chronic diseases worldwide and is associated with a high risk of various cardio-cerebral vascular diseases. It is estimated that approximately 31% of adults worldwide have hypertension, and this proportion is expected to substantially increase to 60% by 2025 (1). Hyperaldosteronism is one of the most common mechanisms of secondary hypertension, which involves an increase in the secretion of aldosterone, because of its function in governing volume and electrolyte homeostasis in the body. Mineralocorticoid receptor (MR) antagonists, mainly spironolactone, have long been used as traditional drugs to control blood pressure but have failed to treat resistant, refractory, and renal hypertension. Therefore, there is a need to enhance knowledge about the regulation mechanisms of aldosterone to identify new therapeutic targets.

Aldosterone is a classic steroid hormone in the renin-angiotensin-aldosterone system (RAAS), which mediates various physiological activities, playing a primary role in the maintenance of water and electrolyte balance by activating MR. MR belongs to nuclear receptor subfamily 3 with glucocorticoid receptor, and the two receptors share the same structure, including the N-terminal domain, DNA-binding domain, and C-terminal ligand-binding domain (2). In general, MR moves from the cytoplasm to the nucleus upon ligand binding to regulate the transcription of its target genes and consequent protein expression. RAAS blockade is a commonly used clinical strategy to alleviate cardiac insufficiency, to inhibit cardiac remodeling, ameliorate proteinuria, and to decrease blood pressure. However, in early 1981, Staessen et al. (3) found that long-term angiotensin (Ang) II suppression with captopril in patients with hypertension did not decrease the plasma aldosterone concentration, which was in contrast to the expectation; in fact, the opposite effect was found in that, despite an initial decrease, the aldosterone plasma concentration increased from 21 to 165 pg/mL after 1 year of captopril treatment. Since then, there has been extensive research effort to better understand this unexpected phenomenon, including exploring the effects of treatment with the Ang II receptor inhibitor candesartan or valsartan in patients with hypertension (4) and the combination of the renin inhibitor aliskiren with valsartan in patients with hypertension and proteinuria (5). However, all these studies consistently supported the same conclusion that long-term suppression with angiotensin-converting enzyme/Ang receptor inhibitors could not effectively reduce the plasma aldosterone concentration and conversely resulted in an increase in the level of circulating aldosterone, which was subsequently defined as the “aldosterone escape” or “aldosterone breakthrough” phenomenon. These findings raised the possibility that other mechanisms are likely involved in stimulating aldosterone secretion independent of RAAS. Studies in the last decade suggest that an autocrine-paracrine mechanism may be responsible for a large part of the aldosterone breakthrough effect. Moreover, the classical receptor MR is only involved in approximately 50% of the activities of aldosterone based on studies employing spironolactone (6, 7), indicating that other aldosterone receptors exist in the cell membrane.

Despite several candidates, these additional receptors remained somewhat of a mystery until the recent discovery of G protein–coupled estrogen receptor (GPER) as a novel aldosterone receptor that primarily mediates its non-genomic actions. Various lines of evidence demonstrate that GPER induces aldosterone biosynthesis (8) and rapid actions, which could help to explain the landmark deviations from expectation emerging from the MR pharmacology studies mentioned above (9). GPER was long considered an orphan receptor before its natural ligand estrogen was discovered. Evidence also indicates that aldosterone likely accelerates cancer growth and spread. Therefore, there has been active research on the use of GPER as a potential therapeutic target for cancers, especially in gynecological malignancies such as breast and endometrial cancers. It is well demonstrated that GPER is required for the proliferation and migration of breast cancer and renal cortical adenocarcinoma cells, which involves the activity of sodium/hydrogen exchanger-1 (NHE-1) and different aldosterone concentrations (9, 10).

**Figure 1:**
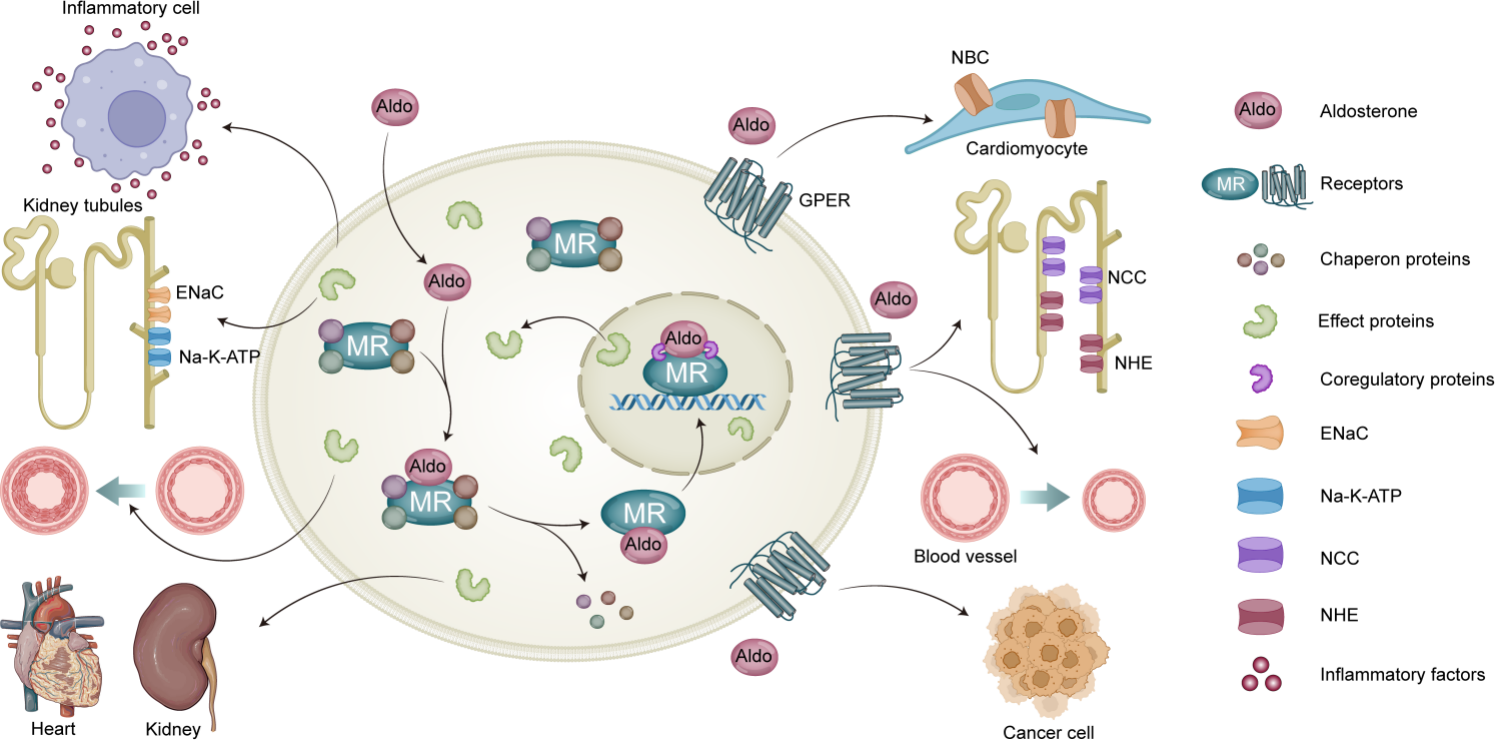
Genomic and non-genomic actions of aldosterone. MR mediates classic genomic signaling of aldosterone, which mainly associated with the expression of Epithelial Sodium Channel (ENaC) and Na,K-ATPase, release of inflammatory factors, vascular growth, as well as renal and heart fibrosis. Aldosterone also mediates non-genomic signaling through GPER activation, which is associated with activity of Sodium/Bicarbonate Cotransporter (NBC), NCC, and NHE; vasocontrictor effect; and development of cancer cell.

Therefore, GPER is emerging as a strong candidate to explain aldosterone regulation and related diseases such as hypertension and cancer. To help advance research in this field and its clinical translation, this review systematically summarizes the mechanisms and actions of GPER activated by aldosterone and elucidates the primary events mediated by GPER following aldosterone exposure (**Figure 1**). Finally, the challenges and prospects in developing GPER as a promising therapeutic target for aldosterone-induced hypertension treatment in the future are discussed, which can help to address the limitations of current treatments.

## 2 Hypertension and aldosterone

Hypertension, including essential and secondary hypertension, is a multifactorial disease associated with an increased risk of various cardio-cerebral vascular diseases. Specific causes of secondary hypertension include aortic coarctation, renal artery stenosis, thyroid disorders, aldosteronism, obstructive sleep apnea, pheochromocytoma, and Cushing syndrome (11). Drug treatment is the first-line therapy for essential hypertension, whereas etiological treatment is more suitable for secondary hypertension. Angiotensin-converting enzyme inhibitors, angiotensin receptor inhibitors, calcium channel blockers, beta receptor blockers, and thiazides are widely used in the clinical treatment for essential hypertension. The guidelines advocate combination drug treatments and emphasize individualized therapy (12). The treatment for secondary hypertension depends on the precise pathogenesis, including inhibition of cortisol secretion in Cushing syndrome, implantation of stents in renal artery stenosis, and other etiological treatments. Moreover, the exciting discovery of a novel vaccine offers a new and practical therapeutic approach to hypertension (13, 14). However, resistant hypertension, the “aldosterone breakthrough” phenomenon, and anti-hypertensive drug intolerance remain challenges in current treatment strategies. Thus, there is an urgent need to identify new drug targets for the treatment of hypertension.

Primary aldosteronism is a common cause of secondary hypertension, which is characterized by hyperaldosteronemia, inducing cardiorenal fibrosis, water and sodium retention, hypokalemia, and vasoconstriction. Aldosterone is distinct from other hormones such as glucocorticoids and androgens in that it regulates electrolyte homeostasis and the extracellular fluid volume. As a significant member of the RAAS, aldosterone is principally synthesized from cholesterol in the zona glomerulosa and is secreted mainly in response to Ang II stimulation, elevated serum potassium levels, and increased adrenocorticotrophic hormone levels (15). Long-term suppression of the RAAS is a widely applied treatment strategy to alleviate high blood pressure but fails to decrease the aldosterone concentration to normal levels. The autocrine/paracrine mechanism is responsible for part of this “aldosterone escape” phenomenon. An increased mast cell population and upregulation of the serotonin 5-Hydroxytryptamine (5-HT) signaling pathway have been observed in aldosterone-producing adenomas (16). Moreover, mast cell degranulation stimulates aldosterone synthesis by releasing 5-HT and activating 5-HT4 receptors (16). In addition to mast cells, chromaffin cells, nerve endings, endothelial cells, and adipocytes regulate aldosterone production by releasing bioactive signals, including neurotransmitters and neuropeptides (17). In recent years, lipid secondary messengers such as phosphatidic acid, diacylglycerol, and sphingolipid metabolites have been implicated in the synthesis and secretion of aldosterone (15).

Unexpectedly, accumulating evidence is revealing that, in addition to MR, aldosterone can bind to GPER to exert its non-genomic actions (9, 18, 19). Furthermore, Ang II can influence aldosterone synthesis through the interaction of Ang II receptor type 1 with GPER (8), providing a mechanism to link GPER overexpression with the elevation in aldosterone in patients with aldosterone-producing adenomas. As a newly discovered receptor responsible for the non-genomic actions of aldosterone, GPER has attracted increasing attention in the context of hypertension owing to its role in vascular reactivity and cardiorenal function.

## 3 GPER: a new receptor for aldosterone

GPER, also known as GPR30, is a seven-transmembrane G protein–coupled receptor (GPCR) that was first identified in breast carcinoma cell lines (20). Similar to classical GPCRs, GPER comprises an N-terminal extracellular domain, seven transmembrane helices linked by three extracellular loops and three intracellular loops, and a C-terminal intracellular domain (21). Mapped on chromosome 7p22, GPER is widely distributed in various mammalian tissues, including the heart, lungs, liver, adrenal glands, intestines, ovaries, and brain, regardless of sex or species (20, 22–24). GPER localizes to the endoplasmic reticulum and plasma membrane (18, 25), thereby signaling from intracellular locations, as observed for some GPCRs (25). GPER can also function in the nucleus and can interact with nuclear steroid receptors, including estrogen receptor α (ERα), ERβ, MR, vitamin D receptor, and glucocorticoid receptor (10, 26–28).

G1 was the first selective GPER agonist identified that activates the receptor with high selectivity and affinity but has little or no affinity for ERα and Erβ (29). Furthermore, G15 and G36 have been classified as selective GPER antagonists (30). Notably, the anti-hypertensive drugs eplerenone and spironolactone were found to partially inhibit G1-mediated extracellular signal–related kinase (ERK) phosphorylation in GPER-expressing cells, suggesting that both drugs act as partial antagonists of GPER (9).

Although originally identified as an orphan receptor (under the name GPR30), a study in SK-BR-3 [SKBR3], a human breast adenocarcinoma cell line, showed that breast cancer cells showed that estrogen is the natural ligand of GPER; the cytomembranes of cells lacking ERα and ERβ expression but expressing GPER showed high-affinity, limited capacity, displaceable, and specific binding to estradiol-17β, and the binding was affected by an increase or decrease in GPER expression with progesterone stimulation and RNA interference, respectively (18). A subsequent study showed that GPER can interact with aldosterone and mediate non-genomic pathways in a manner distinct from the classic MR-mediated activation mechanism. A recent study suggested that the GPER-dependent effects of aldosterone likely occur by direct binding, with a stronger binding potency of GPER to aldosterone than to estrogen; however, the binding of GPER is the strongest to [^3^H] 2‐ME, a high-potency GPER-selective agonist in insect cells without any of the intrinsic mammalian receptors for aldosterone (19).

In general, GPCRs initiate signaling through heterotrimeric G proteins and G protein–independent pathways via G protein–coupled receptor kinase (GRK)–mediated phosphorylation and arrestin coupling (31). Accumulating evidence shows that GPER expressed in cardiac myocytes and fibroblasts can couple to both Gi and Gs proteins to induce epidermal growth factor receptor or insulin-like growth factor-1 receptor transactivation in different tissues (32–34). Although it remains unclear whether aldosterone couples with Gs or Gi when binding to GPER, it is possible that the mechanism is identical to that of the non-genomic activation of estrogen, in which Gs is activated upon binding to GPER, resulting in the stimulation of adenylyl cyclase activity and increased Cyclic Adenosine Monophosphate (cAMP) production in the plasma membranes of SKBR3 and GPER-transfected cells (18, 35). Furthermore, G protein–independent pathways are involved in the non-genomic aldosterone pathway. GRK-2 and GRK-5 are the most abundant cardiac GRKs that phosphorylate GPCRs and non-GPCR substrates (36, 37). GRK2 was suggested to hinder the effects of aldosterone by activating GPER, whereas GRK5 blocks the cardiotoxic MR-dependent effects of aldosterone in the heart (38). Notably, although GPER mediates the non-genomic actions of both estrogen and aldosterone, the effects may differ entirely and could also involve interactions with MR.

## 4 GPER as a main player in non-genomic aldosterone pathways

MR primarily remains in the cytoplasm with chaperones and scaffolding proteins when not bound to its ligands (39). The classic genomic pathway of aldosterone involves binding to MR to cause its nuclear translocation; subsequently, MR and glucocorticoid receptor form homodimers or heterodimers that act as homodimers of hormone response elements on chromosomes, ultimately regulating gene transcription and protein expression (2). Coactivators and co-repressors modulate the transcription of downstream effector proteins (2). The main downstream physiological and pathological effects of this pathway are associated with the expression of the epithelial sodium channel and Na^+^/K^+^-ATPase, release of inflammatory factors, vascular growth, and renal and heart fibrosis (40–42) (**Figure 1**).

Non-genomic events of aldosterone were first identified in 1984, when Moura and Worcel (43) discovered that the rapid aldosterone effects on sodium and potassium excretion in the kidney (44) did not seem to depend on the transcription of genomic information. Since then, various other non-genomic aldosterone-mediated signaling events have been identified, including elevation in intracellular Ca^2+^, inositol 1,4,5-trisphosphate (IP_3_), diacylglycerol, protein kinase C, phospholipase C, and cAMP (39). On the basis of the initial studies, many of the non-genomic actions of aldosterone appeared to be dependent on MR, such as aldosterone-induced ERK1/2 mitogen-activated protein kinase (MAPK) activation in M1 renal cortical collecting duct cells (45) and vascular p38 MAPK and Nicotinamide Adenine Dinucleotide Phosphate (NADPH) oxidase activation via cellular-rabbit squamous cell carcinoma gene (c-Src) (46). Spironolactone was found to be ineffective in inducing the non-genomic events of aldosterone, suggesting that MR may not mediate the non-genomic aldosterone pathways; however, the selective antagonists of MR eplerenone and RU28318 did induce these pathways, indicating that the role of MR in this process could not be ruled out completely (45). Grossmann et al. (47) showed that aldosterone induced the rapid and dose-dependent phosphorylation of ERK1/2 and c-Jun NH_2_-terminal kinase 1/2 in human embryonic kidney cells heterologously expressing MR, which was suppressed by treatment with spironolactone or a MAPK inhibitor but not a protein kinase C inhibitor. However, spironolactone did not inhibit the aldosterone-induced increase in cytosolic Ca^2+^. The authors thus suggested that there are three co-existing aldosterone signaling pathways: (1) a genomic pathway via MR, (2) a non-genomic pathway via MR, and (3) an MR-independent non-genomic pathway (47).

Indeed, studies using the MR antagonist spironolactone have suggested that MR is only involved in approximately 50% of aldosterone’s activities related to sodium-proton exchange and various secondary messengers, including IP_3_ production in different cells (6, 7). Christ et al. (48) demonstrated that membrane receptors rather than MR were responsible for the rapid, non-genomic *in vitro* effects of aldosterone on intracellular electrolytes, cell volume, and the sodium-proton antiporter in human mononuclear leukocytes. In 2011, the involvement of GPER in rapid aldosterone activity was first confirmed, and the significant pharmacological aspects of this action were compatible with the landmark deviations from expectations in MR pharmacology studies (9). The GPER-dependent effects of aldosterone for ERK phosphorylation, mediation of apoptosis, and myosin light-chain (MLC) phosphorylation have also been reported (9).

Currently, the non-genomic aldosterone pathway is well recognized and extensively studied worldwide. The interaction between aldosterone/MR signaling and aldosterone/GPER signaling was first described in vascular smooth muscle cells (VSMCs) by Gros et al. (49), who later found that aldosterone-mediated ERK phosphorylation was inhibited by both eplerenone and G15 in MR-transduced cultured VMSCs or VMSCs transferred with the GPER gene (9). In addition, Ashton et al. (50) examined the non-genomic actions of aldosterone using pegylated aldosterone analog (Aldo-PEG) and showed that activation of GPER alone is not sufficient to cause deleterious effects on myocardial reperfusion injury; however, they further showed that raising reactive oxygen species (ROS) levels may enhance the MR-mediated actions of aldosterone during myocardial reperfusion injury in the heart, elucidating the functional involvement of GPER in rapid aldosterone/MR signaling. The authors concluded that the non-genomic effects of aldosterone did not potentiate the genomic signaling pathways. Further support for this cross-talk comes from experiments showing that aldosterone led to enhanced maximal phenylephrine-induced vasocontraction in mesenteric resistance arteries, which could be reversed by G15, and aldosterone also reduced acetylcholine-induced vasorelaxation in a manner dependent on both MR and GPER (51). In general, the evidence accumulated to date suggests that GPER is more important than MR in the non-genomic aldosterone pathway and that the cross-talk between these receptor interactions further complicates the mechanisms underlying aldosterone-induced events.

## 5 The mediating roles of GPER activation and inhibition in vasoconstriction

The progression of hypertension is associated with the thickening of the vascular wall, increased responsiveness of the vasculature, increased vascular capacitance, and vascular endothelial dysfunction. GPER maps onto chromosome 7p22, a region implicated in hypertension in humans (24), and some studies suggest that deletion of GPER using gene-editing technology could have beneficial effects in lowering blood pressure and improving cardiac function (52, 53). Ogola et al. (54) also demonstrated that deletion of Gper in mice significantly increased pulse pressure but did not protect against hypertension given the lack of cardiac hypertrophy, and there was no difference in systolic and diastolic blood pressure using Gper-knockout and wild-type mice. *In vivo* experiments have demonstrated similar results to studies based on gene-editing technology. Upon estrogen or G1 stimulation, GPER activation tends to show significant cardioprotective effects with differences observed according to sex. Several research groups have independently demonstrated the role of GPER in improving cardiovascular function via endothelial nitric oxide synthase-dependent vasodilation in arterial myography of healthy vessels in postmenopausal women along with an excellent protective effect of GPER in hypertension models (55, 56). Nevertheless, Maruyama et al. (57) claimed that, in a healthy environment, estrogen activity tends to be beneficial (e.g., vasodilation and ROS reduction), whereas, in the context of diseases or risk factors such as hypertension, estrogen may exert adverse effects. Their research revealed that activation of GPER distributed in the rostral ventrolateral medulla in Goldblatt hypertensive rats contributes to sympathetic overactivation, which is associated with the accelerated development of hypertension. The lack of a protective effect of hormone replacement therapy against coronary heart disease in clinical trials also supports the above speculation to some degree (58).

It is now well accepted that aldosterone directly affects the vascular system by inducing oxidative stress, inflammation, hypertrophic remodeling, fibrosis, and endothelial dysfunction. As a newly discovered aldosterone receptor, GPER mediates multiple vascular actions, similar to MR. GPER activation accelerates the progression of apoptosis in VSMCs and vascular endothelial cells (9, 59). In addition, Tang et al. (60) demonstrated that GPER could mediate part of the endothelial inflammatory response induced by aldosterone, suggesting GPER as an alternative target for treating hyperaldosteronism considering the unsatisfactory effect of MR antagonists on cardiovascular risks. Notably, GPER expression diminished after culturing VSMCs, whereas vascular endothelial cells maintained high GPER expression throughout culture. These results were based on adenovirus-mediated GPER gene transfer into VSMCs. Moreover, aldosterone could stimulate smooth muscle–specific MLC phosphorylation via a GPER-dependent mechanism in both newly isolated aortic ring segments and cultured VSMCs. This provided primary evidence that GPER could affect vascular tone (9, 61) because MLC plays a central role in smooth muscle contraction and is determined by the balance of activity between MLC kinase and MLC phosphatase (62). Furthermore, GPER re-expression enhanced aldosterone-mediated contractions in rat aortic VSMCs (61). In addition to the aforementioned direct effects, aldosterone has been shown to enhance the response of vessels to vasoactive substances. Ferreira et al. (51) showed that aldosterone increased the systolic response of mesenteric resistant arteries to phenylephrine, which was prevented in the presence of G15. Furthermore, pre-incubation with aldosterone (10 nmol/L) enhanced the contractile effects of Ang II on human coronary artery by activating GPER (59). The signaling pathways involved in these effects include ERK phosphorylation, PI3K activation, and transactivation of the tyrosine kinase receptor pathways (63).

Thus, it is not difficult to conclude that GPER may activate multiple signaling pathways and have varied effects depending on the cell type and stimulant. However, in contrast to estrogen or G1, aldosterone is more likely to mediate vasoconstriction effects or exert these effects synergistically with other vasoactive molecules. Collectively, this evidence suggests that blocking GPER and MR can attenuate the vascular injury triggered by aldosterone to a great extent. Accordingly, we emphasize that GPER is a promising therapeutic target specifically for aldosterone-induced hypertension rather than for secondary hypertension.

## 6 Role of aldosterone-induced GPER activation in cardio-renal injury

Inflammation, fibrosis, and apoptosis have been reported to be strongly associated with aldosterone-induced events and contribute to end-organ damage in cardiovascular and metabolic diseases. Epithelial sodium channel and Sodium-Potassium Adenosinetriphos Phatase (Na,K-ATPase) are the main downstream effector proteins of MR. Similar to MR, aldosterone also exerts specific effects on podocyte injury and mesangial cell proliferation in the kidney (64). A growing body of evidence suggests that aldosterone regulates various ion channels, including NaCl co-transporter and NHE-1, via GPER to cause water and electrolyte imbalances, thereby deteriorating heart failure or hypertension (65, 66). In addition, increased activity of the sodium/bicarbonate cotransporter is associated with ROS production and Protein Kinase B (AKT) stimulation in rat cardiomyocytes (67). Connection tubule glomerular feedback is a process that increases Na delivery to the connecting tubules, dilating the afferent arteriole and leading to an increase in glomerular filtration rate (68). Ren et al. (67) demonstrated that aldosterone sensitizes the connection tubule glomerular feedback response by acting on NHE-1, which may contribute to renal damage by increasing afferent arteriole dilation and glomerular capillary pressure (glomerular barotrauma). In the brain, it is increasingly appreciated that the nucleus tractus solitarius (NTS), which represents the first central synapse of taste afferent fibers, plays a vital role in controlling fluid and energy balance in response to signals from the periphery and that lesions in this brain region increase sodium intake (69). Qiao et al. (69) found that aldosterone injection into the NTS induces prompt and significant sodium intake, which is suppressed by G15. Furthermore, forskolin-stimulated cAMP levels are elevated in aldosterone-treated cell lines depending on GPER activation (70, 71). Thus, aldosterone may not only influence various ion channels to control water and electrolyte balance but also modulate NTS events associated with changes in cAMP levels, relaying in the parbrachial nucleus before projecting to the amygdala, thereby forming the main neural axis controlling sodium appetite and taste.

However, there is no direct evidence regarding whether aldosterone increases the release of inflammatory factors or promotes fibrogenesis in the heart and kidneys; thus, future studies should focus on these aspects.

## 7 Discussion

Hypertension is a leading preventable risk factor for premature death and disability worldwide, representing a significant global health challenge because of its high prevalence in cardiovascular disease and chronic kidney disease (1). Aldosterone in the RAAS is one of the most critical hormones synthesized by the adrenal gland because it regulates the water and electrolyte balance. Overactivation of the RAAS plays a vital role in the onset and progression of hypertension, mainly through water and sodium retention along with cardio-renal injury triggered by aldosterone. In the classic genomic pathway, aldosterone binds to MR to promote gene transcription and protein expression. MR antagonists, mainly spironolactone, have long been used as traditional drugs to control blood pressure but fail to effectively treat resistant hypertension, refractory hypertension, and renal hypertension. This so-called “aldosterone escape” phenomenon necessitates finding other pathways induced by aldosterone. GPER, a member of the GPCR family, mediates the non-genomic signaling of estrogen and has recently been identified as a new receptor of aldosterone. GPER has been highlighted as a potential therapeutic target for salt-sensitive hypertension in postmenopausal women because activation of GPER could decrease blood pressure in an acute manner in male Sprague–Dawley rats and in a chronic manner in estrogen-deficient female mRen2 rats (55, 72). Gohar et al. (73) recommended GPER as a pronatriuretic factor to control renal Na^+^ handling. However, GPER did not alter blood pressure in intact female mRen2 rats, indicating that GPER may only exert its role in the absence of the endogenous ligand.

According to the National Center for Biotechnology Information nucleotide database, there are three curated mRNA variants of GPER, namely, NM_001505.2 (GPER-v2), NM_001039966.1 (GPER-v3), and NM_001098201.1 (GPER-v4), which share the same coding region and 3′ untranslated region but differ in amino acid accounts, exon–intron organization, and 5′ untranslated regions. In 2021, Pal et al. (74) discovered a new GPER-v5 variant and advocated that more studies on GPER variants are necessary to investigate their role and relevance in physiological and pathological conditions. The GPER P16L variant is a common hypofunctional genetic variant associated with increased blood pressure in women (75). However, no studies have revealed the potential benefits of GPER in men.

Different stimulants may have opposite effects owing to different binding sites or downstream signaling, which is the case for regulatory effects induced by aldosterone and estrogen levels. Estrogen or G1 could trigger vasodilation in the mesenteric arteries involving both endothelial nitric oxide and smooth muscle cAMP signaling via GPER, whereas aldosterone exhibits the converse effect (48, 76). In addition, multiple studies have shown that aldosterone strengthens vasoconstriction and promotes water and sodium retention by activating GPER, which is involved in ERK phosphorylation, the PI3K pathway, and ROS production. The activation of GPER is cardioprotective and decreases blood pressure in the presence of estrogen or G1 stimulation, and these effects are especially evident in postmenopausal women. Nevertheless, aldosterone displays a stronger binding potency for GPER than estrogen; thus, under conditions of primary aldosteronism or hyperaldosteronemia-induced hypertension, inhibiting the activity of GPER would have a better effect on achieving blood pressure control and targeting organ injury. In summary, this review highlights GPER as a potential therapeutic target in aldosterone-induced hypertension, particularly in men and premenopausal women.

## Author contributions

XL (First author): Writing original draft. WK: Conceptualization and review. ZQ: Writing review and editing. ZZ: Writing review and editing All authors have contributed to this review. All authors contributed to the article and approved the submitted version.
